# A 20-Year Review of Biomechanical Experimental Studies on Spine Implants Used for Percutaneous Surgical Repair of Vertebral Compression Fractures

**DOI:** 10.1155/2022/6015067

**Published:** 2022-09-21

**Authors:** Sairam Gajavelli, Aaron Gee, Z. Shaghayegh Bagheri, Emil H. Schemitsch, Christopher S. Bailey, Parham Rasoulinejad, Radovan Zdero

**Affiliations:** ^1^Orthopaedic Biomechanics Laboratory, Victoria Hospital, London, ON, Canada; ^2^Department of Mechanical Engineering, George Mason University, Fairfax, VA, USA; ^3^Kite Research Institute, Toronto Rehab Institute, University Health Network, Toronto, ON, Canada; ^4^Department of Surgery (Division of Orthopaedic Surgery), Western University, London, ON, Canada

## Abstract

A vertebral compression fracture (VCF) is an injury to a vertebra of the spine affecting the cortical walls and/or middle cancellous section. The most common risk factor for a VCF is osteoporosis, thus predisposing the elderly and postmenopausal women to this injury. Clinical consequences include loss of vertebral height, kyphotic deformity, altered stance, back pain, reduced mobility, reduced abdominal space, and reduced thoracic space, as well as early mortality. To restore vertebral mechanical stability, overall spine function, and patient quality of life, the original percutaneous surgical intervention has been vertebroplasty, whereby bone cement is injected into the affected vertebra. Because vertebroplasty cannot fully restore vertebral height, newer surgical techniques have been developed, such as kyphoplasty, stents, jacks, coils, and cubes. But, relatively few studies have experimentally assessed the biomechanical performance of these newer procedures. This article reviews over 20 years of scientific literature that has experimentally evaluated the biomechanics of percutaneous VCF repair methods. Specifically, this article describes the basic operating principles of the repair methods, the study protocols used to experimentally assess their biomechanical performance, and the actual biomechanical data measured, as well as giving a number of recommendations for future research directions.

## 1. Introduction

**Figure 1:**
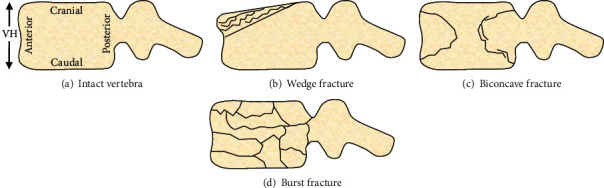
VCF subtypes. VH is vertebral height. Some burst fractures result in bone fragments impinging into the spinal canal space, which is not shown here. Drawings are not to scale.

A vertebral compression fracture (VCF) is an injury to a vertebra of the spine (Figure 1). VCFs are type A fractures according to the Orthopaedic Trauma Association (OTA)/AO spine classification system [1]. There are 3 main versions [1, 2]. A VCF “wedge” fracture is the most common, whereby only the anterior portion of the vertebra is compressed. A VCF “biconcave” (or “split”) fracture occurs, in which the middle section of the vertebra is cracked and collapses while outer walls remain intact. A VCF partial or full “burst” fracture is the least common, whereby the vertebra is crushed into multiple small fragments.

The most frequent underlying risk factor for experiencing a VCF is osteoporosis [2]. In 2010, 53.6 million US adults over 50 years of age were affected to some degree by osteoporosis [3]. As such, 20% of those above 70 years of age and 40% of those above 80 years of age will experience a VCF [2, 4]. Moreover, postmenopausal women are perhaps at the greatest risk for a VCF due to a notable decrease in bone density [2, 4]. An initial VCF also increases the risk by 20% of a subsequent VCF [2, 5]. Consequently, in the US alone, about 700,000 VCFs occur annually [6].

VCFs have the following clinical consequences [7]. There is a loss of vertebral height (VH), kyphotic deformity, altered stance, and back pain. Also, there is reduced mobility (causing pressure sores, infection, venous stasis, and disuse atrophy), reduced abdominal space (causing gastrointestinal problems), and reduced thoracic space (causing respiratory and pulmonary problems). As such, there is a 40% increased mortality rate after 8 years for VCF patients vs. uninjured age-matched patient controls. So, mechanical stabilization of the VCF in order to achieve physical restoration of VH is of great importance to prevent or minimize the clinical complications listed above.

VCFs were once traditionally addressed by, and sometimes still can be handled using, nonsurgical treatment with narcotics, orthotics, bed rest, and time; however, bed rest can further increase pain by accelerating bone loss and muscle deconditioning [8]. Therefore, vertebroplasty (VP) is a percutaneous surgical technique invented in France in the 1980s, which involves injecting cement into the affected vertebra for mechanical stability [9–12]. VP was initially used to treat vertebral lesions, it was later adapted to address VCFs, and it was the benchmark treatment for many years to treat VCFs. Over the past several decades, newer percutaneous surgical techniques have been developed for addressing VCFs that potentially provide better biomechanical stability and clinical outcomes, such as kyphoplasty (KP), stents, jacks, coils, and cubes [7]. Consequently, more than $990 million are spent annually in the US alone on surgically treating VCFs [2].

There have been several previously published review articles that deal with percutaneous VCF repair methods. Filippiadis et al. [7] focused their review on clinical outcomes with very few remarks on biomechanics. Wilcox [11] published a biomechanical review of experimental and computational modeling work, but it was almost 2 decades ago and only examined VP. Badilatti et al. [12] reviewed biomechanical computational models that only studied the VP method. Martin-Lopez et al. [13] conducted a review of stent fixation of VCFs, but only for clinical assessments of VH, pain, and function without any biomechanical content.

In contrast, the present article provides a 20-year review that focuses on (a) biomechanical parameters rather than clinical outcomes, (b) experimental testing as the “gold standard” to which even computational studies must eventually be compared, and (c) at least 1 important paper representative of every known traditional and newer percutaneous VCF repair technique. This article will describe each VCF repair method's basic operating principles, general findings about the experimental protocols used and the biomechanical outcomes measured, and recommendations for future research directions.

## 2. Basic Operating Principles of VCF Repair Methods

**Figure 2:**
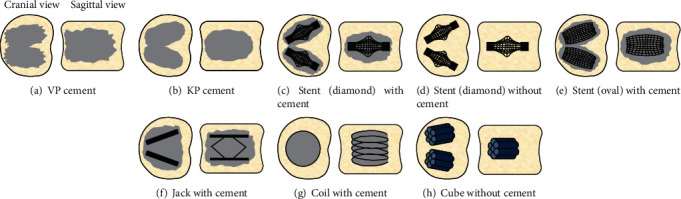
Percutaneous surgical repair methods for VCFs. Cement is represented by gray areas. Fracture lines and posterior bony elements are not shown. Drawings are not to scale.

It is important, first of all, to understand the basic design and function of all known percutaneous treatments and implants for addressing VCFs (Figure 2).

*Vertebroplasty* (VP) is the oldest technique that surgeons have used. It is conducted under fluoroscopy to guide a bone biopsy needle posteriorly into the vertebra via an extrapedicular posterolateral, transpedicular, unipedicular, or bipedicular approach (Figure 2(a)) [7, 9–12, 14–20]. The needle is then used to inject a volume of bone cement (e.g., polymethyl methacrylate (PMMA)) in order to fill the cracks and gaps in the vertebra, promote some restoration of VH due to the natural diffusion and expansion of the cement, and enhance mechanical stability of the injured vertebra.

*Kyphoplasty* (KP) was developed later to have more control over cement injection and to maintain better VH restoration compared to the older VP method. This technique is done using fluoroscopy to insert a bone biopsy needle posteriorly through both pedicles of the vertebra (Figure 2(b)) [7, 16, 18–33]. The needle is removed, guidewires are inserted, tubes are passed over the guidewires, and balloons (i.e., bone tamps) are inserted through the tubes. The balloons are inflated to decompress the vertebra and restore VH, while creating a cavity inside the vertebra. Finally, the balloons are deflated and removed, and a needle is used to inject cement into the cavity.

*Stents* are cylindrical mesh tubes made of steel, titanium, or cobalt-chromium alloy that can potentially reduce or eliminate fractures caused by stress concentrations around the overly rigid cement ball made by VP and KP, as well as the sharp edges of jacks (Figures 2(c)–2(e)) [7, 13, 21, 23, 27, 30, 31, 34]. In particular, under fluoroscopy, a surgical drill is used to create a thin channel posteriorly through both pedicles into the vertebra. Then, stents are inserted into the channels and expanded symmetrically in all directions into a diamond-shape using a mechanical actuator or into an oval-shape using an inflating balloon, depending on the stent design. Next, the actuator or balloon is removed. Finally, a needle is used to inject cement for additional mechanical stability for all oval-shaped stents, but cement may or may not be required for diamond-shaped stents because they are better able to retain their expanded shape.

*Jacks* are metal 4-sided link-hinge devices that can better maintain VH compared to traditional VP (which cannot fully restore VH due to cement leakage) or KP (which loses some restored VH after balloon removal) (Figure 2(f)) [7, 24, 25, 28, 29, 35–37]. Assisted by fluoroscopy, a bone biopsy needle is inserted posteriorly through each pedicle, a guidewire is inserted, the needle is removed, and a reamer is passed over the guidewire into the vertebra to ream out space for the implants. The jacks are then inserted into each cavity and then expanded in the cranial-caudal direction to the desired extent. Finally, a needle is used to inject cement to surround and improve the mechanical stability of the jacks, which remain permanently. The jack ostensibly is able to provide 500 to 1000 N of load support in the cranial-caudal direction.

*Coils* are hollow helical tubes made of PEEK (poly ether ketone) that can substantially reduce the amount of cement required and do not require the creation of a cavity in the vertebra compared to KP, stents, jacks, or cubes (Figure 2(g)) [7, 32]. Under fluoroscopy, a trocar is used posteriorly to create a unipedicular canal through which a guidewire is inserted as a continuous preshaped loop into the vertebra. A single hollow coil is then deployed over the guidewire into the vertebra until the desired VH restoration is achieved, after which the guidewire is retracted. Finally, cement is injected into the hollow coil through a delivery system, until the cylindrical column of cancellous bone enclosed by the coil is filled with cement.

*Cubes* are bundles of hexagonal hollow tubes that are fabricated from brass, steel, or titanium which crudely simulate the porous architecture of cancellous bone, thereby eliminating or reducing the need for cement, providing a scaffold for cancellous bone regeneration, and reducing the stress concentrations associated with VP, KP, stents, jacks, and coils (Figure 2(h)) [38–41]. These “porous” cubes are inserted into the vertebra through 1 or both reamed or drilled pedicles or, alternatively, through the hollow canal of a pedicle screw. The cube implants remain permanently to give ongoing structural support to, and decompression of, the injured vertebra. A small amount of cement may occasionally be used to fill gaps around the implant and provide additional support.

## 3. General Findings from Biomechanical Studies

### 3.1. Overall Trends

**Figure 3:**
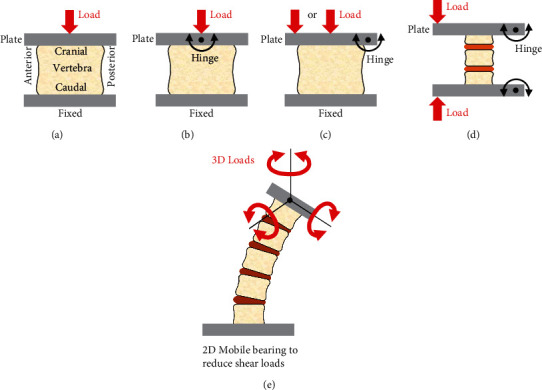
Typical experimental test set-ups used to study the biomechanics of percutaneous VCF repair methods. (a) Parallel plates, (b) pivoting plate with 1D or 3D center hinge, (c) pivoting plate with 1D edge hinge, (d) 2 pivoting plates with 1D edge hinges, and (e) 6-degree-of-freedom spine tester. Fracture lines and posterior bony elements are not shown. Diagrams are not to scale.

All biomechanical studies reviewed here are summarized with details on donor demographics, experimental test protocols, primary biomechanical results, and secondary biomechanical outcomes (Figure 3, Table 1) [14–35, 37, 38, 40, 41]. Several trends were noted.

Firstly, almost all studies used the same experimental testing sequence, although details differed. Specifically, a specimen was prepared as a multivertebral spine or an isolated vertebra. Mechanical testing was then done to get a baseline value for the intact specimen. A complete fracture was then made in the vertebra of interest, which was then fixed using a VCF repair method. Mechanical testing was repeated on the repaired specimen, and the final result was recorded.

Secondly, VP and KP have a longer multidecade history of clinical use than the new stents, jacks, coils, or cubes; therefore, most reports used VP and/or KP as the “gold standard” control [21, 23–25, 27–32, 37, 38, 40, 41]. But, some studies optimized a particular repair method under different conditions, but without comparing it to another technique [14, 15, 17, 20, 22, 26, 33–35].

Thirdly, there was a wide range of numerical values for stiffness, failure load, and restored VH for a given repair method and between repair methods because of the variations in donor demographics and test protocols (Figure 3, Table 1).

Fourthly, in 14 of 14 studies, newer implants like stents, jacks, coils, or cubes performed equivalently to, or better than, traditional KP for the measured outcomes, suggesting their potential clinical benefits [21, 23–25, 27–32, 37, 38, 40, 41].

Fifthly, compared to all other repair techniques reviewed (Table 1), the diamond-shaped stent gave the highest absolute values for stiffness and failure load [34], while the oval-shaped stent achieved the greatest VH following repair with respect to prefracture intact levels [27]. But, this trend needs to be confirmed by a direct comparison of all the techniques in the same study under the same experimental conditions, which has not been done to date.

Sixthly, cement volume did not have much influence on results for implants after a minimum cement volume was injected [24, 28, 35], while adding bone graft to an implant did not necessarily improve its properties [34]. This implies that an implant's inherent mechanical stability was more important than additional augmentation.

Finally, in the rest of this section, the terms “better,” “different,” “statistically different,” etc., mean that pairwise comparisons of biomechanical data between test groups were statistically significant, i.e., *p* < 0.01, 0.05, etc.

### 3.2. The VP Method

VP has been assessed by 4 studies for inherent biomechanical characteristics in an attempt to optimize it, but without comparing it to other methods [14, 15, 17, 20]. Concerning methodology, they had several commonalities, such as (a) the injection of PMMA-based cement, (b) the use of human cadaveric vertebrae from thoracic and/or lumbar regions, and (c) the creation of an initial anterior wedge fracture via uniaxial static compression that was then repaired using VP just before final mechanical testing [14, 15, 17, 20]. But, the many differences make interstudy data comparison difficult, such as (a) mostly female vertebrae [14, 15, 20] vs. almost equal gender representation [17], (b) osteopenic or osteoporotic bone [14, 15, 20] vs. unknown bone quality [17], (c) 2 parallel plates for applying load [14, 15, 20] vs. a 3D pivoting plate [17], (d) uniaxial static compression and/or bending for the final test [15, 17, 20] vs. uniaxial cyclic compression [14], and (e) measuring plate-on-bone contact stress to determine any stress riser effects due to the cement bolus inside the vertebrae [17] vs. not measuring contact stress [14, 15, 20]. Even so, results showed no difference in stiffness for VP when using 3 cements having different chemical additives, although no failure loads were measured [14]. In contrast, the other reports showed VP had higher stiffness and failure load when using a particular cement compared to other brands that had different chemical formulations [15] or lower Young's modulus [17]. Moreover, images from pressure-sensitive film inserted at the plate-on-bone interface showed higher peak contact stresses and larger contact areas when VP employed cement having standard vs. low Young's modulus [17]. Furthermore, there was no difference in the ability of 3 different cements after VP treatment to partially restore VH to prefracture levels, but VH was lost again after each of several uniaxial cyclic compression sequences [14]. Finally, the bipedicular and the unilateral posterosuperior approaches to inserting cement vs. the unipedicular approach resulted in greater compression stiffness, compression failure strength, right bending stiffness, and VH restoration, but not stiffness in flexion, extension, or left bending [20]. Based on these results, the biomechanical performance of VP is particularly affected by the type of cement employed, as well as the angle and location of cement insertion.

### 3.3. The KP Method

KP has been characterized by 3 investigations for its inherent biomechanical behavior under different conditions, but without comparison to other repair methods [22, 26, 33]. Methodologically, the studies had little overlap: (a) human cadaveric thoracic and lumbar vertebrae and (b) male and female donors [22, 26]. However, there were many variations in experimental protocol, such as (a) long specimens composed of 5 vertebrae [22] rather than isolated vertebrae [26, 33], (b) whole vertebrae [22, 26] rather than cylindrical sections removed from the middle of the vertebrae [33], (c) osteoporotic or osteopenic bone [26] rather than unknown bone quality [22, 33], (d) a 6-degree-of-freedom spine tester to apply realistic 3D moments [22] rather than a pivoting plate or 2 parallel plates to apply static compressive load [26, 33], and (e) the initial creation of a burst or anterior wedge fracture [22, 26] rather than an unfractured specimen [33] that was then treated using KP prior to final mechanical testing. Nevertheless, results showed no difference in range of motion (as a surrogate for inverse stiffness) for multivertebra spines that were intact vs. repaired by KP for forward/backward bending, left/right bending, or left/right torsion, while surface bone strains remained very low indicating a small risk for refracture [22]. Also, after KP repair of isolated specimens, there were no statistical differences between PMMA cement alone vs. non-PMMA cement with or without polymer fiber reinforcement for stiffness [26, 33], failure load [26], or cement volume required [26], whereas all cement groups may [26] or may not [33] provide higher stiffness and failure load compared to uncemented controls. Based on these data, KP treatment of fractured and unfractured specimens can replicate the biomechanical properties of intact controls regardless of the type of cement.

KP has also been compared by several studies to the original VP method to ensure that it is a valid alternative treatment [16, 18, 19]. From the standpoint of testing protocol, the studies had few similarities, like (a) human cadaveric specimens from the thoracic and lumbar regions and (b) specimens that were all over 50 years of age [16, 18, 19]. In contrast, methodological variations may account for any contradictory results, such as (a) all female specimens [16] vs. a mixture of genders [18, 19]; (b) osteoporotic bones [16] vs. a mixture of osteoporotic, osteopenic, and normal bone [18] vs. unknown bone quality [19]; (c) uniaxial static compression through 2 parallel plates [16, 18] vs. static plus cyclic compression via a pivoting plate [19]; and (d) the initial creation of an anterior wedge injury [16, 19] vs. unfractured vertebrae [18] that would then be treated using KP or VP prior to final mechanical testing. Even so, some of these studies showed no differences between KP and VP in stiffness [16, 19], failure load [18], VH after final mechanical testing [18], or cement volume used [16, 18]. However, contradictory results, likely due to methodological differences, showed VP could have a higher stiffness [18] and failure load [16], whereas KP restored more VH immediately following treatment and/or maintained more VH after final mechanical testing [16, 19]. Moreover, after final mechanical testing, fracture lines were located above and below the cement (VP, 37% of cases; KP, 74% of cases), only above the cement (VP, 8%; KP, 8%), and only below the cement (VP, 55%; KP, 18%) [18]. Based on these results and KP's greater ability to control cement injection via inflatable balloons, it is an acceptable alternative to the older VP method. Consequently, most contemporary biomechanical evaluations use KP as the control group as discussed below, rather than the older VP technique.

### 3.4. The Stent (Diamond) Method

There was 1 biomechanical report which characterized the inherent properties of cementless diamond-shaped stents without comparison to other repair techniques [34]. This implant was developed to address potential problems of cement augmentation during vertebral repair like cement leakage, pulmonary emboli, and stress risers. Researchers induced osteoporosis in 12 live female sheep, created a standardized osteotomy to simulate a burst fractures in L2, and then repaired the fractures using a cementless stent with or without additional bone autograft for support. Sheep were euthanized, repaired L2 and intact L3 vertebrae were removed, and quasistatic compression was applied at 2 mm/min to the vertebrae using 2 parallel plates until fracture or 16 kN was reached. After surgery but before mechanical testing, the stents with bone autograft restored kyphosis angle better than stents without bone autograft, whereas the opposite was the case regarding VH restoration; however, it is unclear if these differences were actually statistically significant. After mechanical testing, there were no differences in stiffness or failure load between the stent groups or with respect to the intact control. Based on these results, cementless diamond-shaped stents with or without bone autograft appear to restore the biomechanical properties of fractured vertebrae to prefracture levels.

Diamond-shaped stents have also been compared biomechanically to the standard KP treatment to validate their performance [23, 30]. A number of methodological similarities existed between these studies probably because they were performed by the same research group, such as (a) human cadaveric vertebrae from the thoracic and lumbar regions, (b) older bone specimens averaging 68 or 77 years of age, (c) unknown bone quality since clinical *T*-scores were not reported; (d) the initial creation of anterior wedge fractures which would then be repaired with a titanium stent prior to the final tests; and (e) a pivoting plate to apply static compressive loads [23, 30]. The only minor divergence between the studies was the use of male and female donor specimens [23] instead of only male donor specimens [30]. One of the studies showed no differences between stents without cement, stents with cement, or KP for stiffness, yield load (i.e., the load at the onset of plastic deformation), failure load (i.e., the greatest load achieved), and the amount of cement volume required, but VH was not measured [23]. Similarly, the other investigation on stents with cement vs. KP showed no differences for stiffness, yield load, or failure load, whereas the stent required notably less cement volume and maintained anterior VH better after mechanical testing than KP [30]. Based on the outcomes, diamond-shaped stents with or without cement biomechanically perform the same or better than the traditional KP technique.

### 3.5. The Stent (Oval) Method

There are 3 reports on the oval-shaped stent implant, whereby it was biomechanically validated against a KP control [21, 27, 31]. Regarding methodology, there were a few similar aspects, like (a) the use of both male and female human cadaveric specimens, (b) older donors that were at least 55 years old, (c) vertebrae from the thoracic and lumbar regions, and (d) applying static compression by 1 or 2 pivoting plates to create an initial anterior wedge fracture that was then repaired using an oval stent with cement or KP just prior to final testing [21, 27, 31]. But, there were also divergences in protocol which could account for any conflicting results, such as (a) a mixture of osteoporotic, osteopenic, and normal bones [27] vs. unknown bone quality [21, 31]; (b) multivertebrae specimens [21] vs. isolated vertebrae [27, 31]; and (c) a 6-degree-of-freedom spine tester [21] vs. a pivoting plate [27, 31] to perform the final tests. Notwithstanding, multivertebrae specimens under flexion/extension bending showed no differences between oval stents vs. KP for range of motion (as a surrogate for inverse stiffness) or kyphosis angle (as a surrogate for VH) for intact, fractured, or repaired specimens [21]. Similarly, isolated vertebrae under cyclic compression and then static compression until failure showed no differences between oval stents vs. KP for stiffness and failure load [27, 31]. Oval stents restored VH better immediately after fracture repair (but prior to final mechanical testing) vs. KP, which experienced more “deflation” after balloon removal [27, 31]. All studies showed that an equivalent amount of cement volume was required for oval stents and KP [21, 27, 31]. Based on these findings, oval stents provide similar or enhanced biomechanical performance vs. KP.

### 3.6. The Jack Method

There was 1 report which attempted to optimize the performance of the jack device under different conditions, but without validating it against other repair methods [35]. This investigation assessed the influence of 61% partial cement filling vs. 100% full endplate-to-endplate cement filling in a cadaveric investigation. Donor demographics for gender and age were not given, but all specimens were osteoporotic. The 21 intact vertebrae (13 thoracic, 8 lumbar) were each loaded in quasi-static compression at 5 mm/min using an eccentric external load to create anterior wedge fractures with a reduced anterior VH by 35%. After repair by the jack using partial or full cement filling, VH restoration with respect to intact values was statistically the same for both groups. After mechanical testing using the same protocol as above, stiffness was statistically equivalent between partial and full cement filling groups, but the full cement filling group had a 1.49x higher failure load. Based on the above data, it is recommended that jacks be used with full cement filling in order to achieve the best biomechanical results.

Jacks, as a relatively new method, have also been validated directly against traditional KP by several research teams [24, 25, 28, 29, 37]. These reports had some overlap in experimental protocol, such as (a) the use of human cadaveric vertebrae from thoracic and lumbar regions and (b) the application of static compressive load using a pivoting plate to initially create an anterior wedge fracture which is then repaired prior to final testing [24, 25, 28, 29, 37]. But, there were some inconsistencies in methodology that make direct data comparison difficult, such as (a) various age ranges of 51 to 69 years [28], 60 to 70 years [25], 55 to 80 years [37], 79 to 93 years [24], or unknown ages [29]; (b) the use of all female bones [24, 25, 37], all male bones [28], or unreported gender [29]; (c) all osteoporotic vertebrae [24, 25, 29, 37] rather than all normal vertebrae [28]; (d) only subjecting specimens to static compression for all tests [25, 29] compared to a combination of static and/or cyclic compressive loading [24, 28, 37]; and (e) isolated vertebrae [24, 25, 28, 29] rather than multivertebral specimens [37]. Nevertheless, results showed the jack had the same stiffness [28] and failure load [28, 29] compared to the standard KP technique, regardless of the amount of cement used to augment the jack. Moreover, the jack achieved the same [28, 29, 37] or a greater [24, 25, 28, 37] amount of VH restoration immediately following treatment and/or after final mechanical testing vs. KP depending on the amount of cement used for the jack. In this regard, reports were contradictory on the cement volume required for surgical repair according to the manufacturer, whereby some showed the jack required equal [37], less [29, 37], or more [25] cement than KP depending on the vertebral location. Based on these observations, the jack generates equal or superior biomechanical properties vs. traditional KP.

### 3.7. The Coil Method

Only 1 team of investigators to date has assessed the coil implant's biomechanical performance, whereby they compared it to the traditional KP method [32]. Donor demographics included gender (4 male, 5 female), age range (58 to 87 years), and bone quality (100% osteoporotic). The 14 triple-vertebra intact specimens (T9-T11, T12-L2, and L1-L3) were quasistatically compressed at 1 mm/s with 2 pivoting plates until anterior VH of the middle vertebrae T10, L1, and L2 were reduced by 50%, thus creating an anterior wedge fracture. Intact, fractured, and repaired specimens were each loaded quasistatically from 0 to 600 N, then cyclically from 200 to 500 N for 50,000 cycles at 3 Hz, and finally quasistatically again. There were no statistical differences between the coil and KP for stiffness, displacement, or VH for the intact, fractured, repaired, or postcycling conditions. However, the coil required 2.9x less cement volume injection and reported no cases of cement leakage compared to KP. No failure loads were measured or reported by these investigators. Based on these outcomes, the coil device is able to provide the same or better biomechanical properties vs. the standard KP procedure.

### 3.8. The Cube Method

Only 3 biomechanical evaluations of “porous” cube implants have been published to date [38, 40, 41]. The only commonality in experimental protocol was the use of a traditional VP and/or KP as the control group. But, there were many disparities in protocol, such as (a) human cadaveric vertebrae [40, 41] compared to an in vitro sheep model [38], (b) vertebrae from thoracic and lumbar regions of the spine [40, 41] rather than unspecified locations [38], (c) osteoporotic specimens [40] rather than unknown bone quality [38, 41], (d) male and female specimens [41] compared to unreported gender distribution [38, 40], (e) multivertebrae specimens [40, 41] vs. isolated vertebrae [38], (f) the initial creation of an anterior wedge injury [40, 41] instead of just notching the anterior wall to mimic a pending fracture [38] that was then repaired prior to final mechanical testing, and (g) a test jig that applied physiological-type cyclic bending moments [40, 41] vs. 2 parallel plates that applied uniaxial static compression [38] for final testing. Nevertheless, there were no differences in range of motion (as a surrogate for inverse stiffness) or VH loss between titanium cubes without cement, KP, or VP after each of 3 forward/backward cyclic bending sequences of increasing moment [41]. Also, cemented titanium cubes had a range of motion for all bending and torsion tests that was higher than uncemented titanium cubes that had additional rod fixation/fusion, although there was no difference with respect to VP [40]. Similarly, brass vs. steel cubes plus cement were equal in stiffness to each other, both metal cubes plus cement had higher stiffnesses than VP and KP controls, and steel cubes plus cement had larger failure loads than all other groups [38]; however, no proper statistical analyses were done possibly because of small sample size; thus, these differences may potentially be artefacts. Based on these numerical data, the cube implant can provide equivalent or superior biomechanical performance to the traditional VP and KP repair techniques.

## 4. Future Directions for Biomechanical Studies

### 4.1. Standardized Testing

The studies reviewed above have variations in experimental protocols typical of biomechanical studies (Figure 3, Table 1): (a) inconsistencies in donor demographics regarding age, gender, and bone quality; (b) the use of individual vertebrae or longer multivertebrae spine segments; (b) various test apparatuses for load application through pivoting plates or 6-degree-of-freedom spine testers; (d) different endpoints for the final VH that is achieved during initial fracture creation, as well as the type of fracture made; (e) quasitatic and/or cyclic loads; and (f) a variety of biomechanical outcome parameters. As such, a proper direct comparison between studies can sometimes be extremely difficult, if not impossible. Future researchers could consider implementing widely used and standardized experimental protocols that involve using multivertebrae spines and 6-degree-of-freedom apparatuses that apply pure moments to more realistically simulate forward/backward bending, left/right bending, and left/right torsion [42–46]. This would make proper interstudy comparisons easier and the results more clinically relevant, although there are practical limitations that researchers often face, such as budget restrictions, availability of longer spine specimens, and access to advanced 3D spine testers.

### 4.2. Bone Demographics

There is a wide variety in the demographics of the vertebral specimens used in the studies reviewed with respect to gender, age, and bone quality. But, it is known that the majority of clinical cases of VCF happen in elderly patients above 70 years old, especially postmenopausal females who are more prone to osteoporosis as a risk factor for VCF [2, 4]. Some of the reviewed studies, however, used donor vertebrae that were only male [28, 30] that had some younger specimens aged in the 40s, 50s, or 60s [14–18, 21, 27, 28, 32] and that had no or few bones with osteoporosis or whose bone quality was unreported properly to determine if osteoporosis was present [17–19, 21–23, 28–31, 33, 41]. Future researchers are encouraged, therefore, to procure older female bones and/or osteoporotic bones for their VCF studies to make the findings more clinically useful.

### 4.3. Biconcave Fractures

No studies to date have experimentally examined the biomechanics of biconcave (or split) VCF repair, likely because these injuries are less common than anterior wedge fractures. Granted, in vitro creation of a biconcave injury by plate compression may be much more difficult practically than an anterior wedge or burst injury. However, it may be possible to do so by careful osteotomy using surgical tools or industrial machine shop equipment. It is important for some researchers to do so, since biomechanical results from an anterior wedge or burst fracture repair may not be generalizable to a biconcave injury due to its unique fracture geometry which will result in different stress distributions on the bone, cement, and implant.

### 4.4. Coil Implant Studies

Only 1 study to date experimentally examined the coil implant from a biomechanical viewpoint [32]. The focus of that study was to compare the coil to standard KP for basic biomechanical properties like stiffness and VH restoration, as well as cement volume injected and cement leakage. Thus, additional work could be done on the coil regarding failure load, stresses on the host vertebra and implant, stresses on adjacent vertebrae and discs, and the coil design by varying its material properties and geometry.

### 4.5. Cube Implant Studies

Few investigations to date experimentally analyzed “porous” multitube cube devices from a biomechanical standpoint [38, 40, 41]. One of these studies primarily examined the influence of cube shape and material on bone stress distribution [38], which was followed up with a computational biomechanics analysis that looked at the effects of tube diameter and thickness [39]. However, further work could still be done evaluating tubes with varying directions or distributions to more accurately replicate the anisotropy of cancellous architecture, the total number of tubes per cube device, the total number of cube devices inserted into the vertebra, and even the injection of some cement for additional support.

### 4.6. Direct Comparison of All VCF Repair Techniques

The vast majority of the studies reviewed compared the biomechanical behavior of a particular device to one of the older more established “gold standard” techniques like VP or KP as a way to gage that implant's relative performance. Alternatively, some investigators changed various parameters in using a given implant (e.g., cement volume) to optimize the use of that implant. However, no investigation has directly compared all the known VCF fixation methods (i.e., VP, KP, stents, jacks, coils, and cubes) in the same study under identical biomechanical testing conditions to more definitively determine their relative pros and cons. Although performing such a larger study may require more finances, personnel, resources, and time, it would help to have a more objective evidence-based way to choose which implant is best for their VCF patient. Similarly, no team has directly compared any 2 of the newer implants (i.e., stents, jacks, coils, or cubes) to one another in the same experimental biomechanical investigation; this may be an easier task than the larger study suggested.

### 4.7. Fracture Micromotion

The inherent assumption in all the studies reviewed is that a VCF repair method should result in the highest possible mechanical stiffness in order to more effectively and quickly stabilize the vertebral injury. This is indicated by the use of materials that have a much higher Young's modulus *E* than vertebral cancellous bone (*E* = 0.03 to 1 GPa), such as PMMA cement for VP and KP (*E* = 2.5 GPa) and PEEK for coils (*E* = 3.5 GPa), as well as titanium alloy (*E* = 106 to 155 GPa), stainless steel (*E* = 200 to 230 GPa), and cobalt-chromium alloy (*E* = 210 GPa) for stents, jacks, and cubes [39, 47]. However, biomechanical research into long bone fractures has increasingly established that a small amount of controlled axial micromotion at the fracture site of 2 to 10% strain actually enhances secondary-type fracture healing via early callus formation [48]. In contrast, shear micromotion at the fracture site can have a negative influence on callus formation if the ratio of shear to axial micromotion is >1.6 [49]. Consequently, new strategies (e.g., fiber-reinforced composite materials and dynamized implants) that are more mechanically flexible than traditional metal implants are being investigated for long bone fracture repair [47, 50–52] and even spine fixation/fusion [47, 53–55]. Yet, no reports to date have investigated the effects of this phenomenon specifically for the percutaneous VCF repair methods discussed presently by designing more flexible implants; this may be a new area of research.

### 4.8. Computational Modeling

Although finite element computational studies of the biomechanics of VCF repair was not the focus of this review, some remarks should be made. Great improvements in computational tools in recent years have encouraged some researchers to utilize finite element models, rather than costly experiments to investigate VCF treatments [12, 39, 56–68]. However, the best way to model VCF fixation is still a controversial topic. For instance, the shape of the cement bolus can be modelled in different ways [12, 68]: (a) an idealized barrel, ellipsoid, spheroid, or torus; (b) the shape of the host vertebra itself; or (c) realistic cement shapes based on medical imaging scans of patients with injured vertebrae that have been treated with cement. The vertebral volume that is filled with cement can also range widely from partial to complete filling [12]. The incision location and insertion angle can be simulated for a bone biopsy needle during cement injection or for a drill bit, reamer, or implant expander during implant insertion [12]. The behavior of cement flow using models with different fluid mechanics and dynamics can take into account elements like pressure, viscosity, flow rate, orifice shape, and bone marrow displacement in the porous cancellous network [12]. The cancellous region around the cement and/or implant can often be compacted during VCF treatment, which can alter the mechanical stresses on surrounding structures [60]. The vertebral bone is commonly modeled as osteoporotic to mimic older patients [65–68], but normal bone quality can also be easily simulated if younger patients are of concern. The shape, size, and material of the implant itself can be optimized [39, 64], while the implant can be used alone or combined with other spine fusion devices [65]. The amount of restored VH following VCF treatment can be modeled as partial or complete [65]. All these factors, and others, can not only influence the reliability of the predicted mechanical stresses on the cement and implants but also on surrounding structures like the treated vertebrae's cancellous bone, cortical bone, and endplates, as well as adjacent discs and vertebrae. Of particular concern may be VCF repairs that generate stress risers causing refracture and/or stress shielding causing bone loss. Consequently, the reliability of VCF finite element models eventually needs to be confirmed by validation experiments similar to those reviewed in this article, as done successfully by some authors [59]. However, computational modeling of VCF repair methods has not and perhaps cannot adequately simulate a patient's subjective experience of pain, movement, and functionality.

## 5. Conclusions

VCFs are most commonly found in people with osteoporosis. The clinical consequences of this fracture for many years were percutaneously surgically treated using simple bone cement injection using the VP technique. However, VP cannot fully restore VH and permits cement leakage, necessitating the development of newer percutaneous techniques like KP, stents, jacks, coils, and cubes. This article provided a review of experimental biomechanical studies that have evaluated these repair methods. In particular, this review described the basic operating principles of the repair methods, the study protocols employed to experimentally evaluate their biomechanical performance, and the biomechanical data that were measured. Finally, several practical recommendations were made for future experimental research to help make studies more clinically relevant and easily comparable to one another.

## Figures and Tables

**Table 1:** Summary of experimental biomechanical studies on percutaneous VCF fixation. Stiffness and failure data are for repaired vertebrae after final mechanical testing, but VH data are for repaired vertebrae before final mechanical testing. All studies were done on human cadaveric vertebrae, except for those using pig [33] or sheep [34, 38] vertebrae.

	VP	KP	Stent (diamond)	Stent (oval)	Jack	Coil	Cube
Studies	[14–20, 38, 41]	[16, 18–33, 37, 38, 41]	[23, 30, 34]	[21, 27, 31]	[24, 25, 28, 29, 35, 37]	[32]	[38, 40, 41]
Gender	M, F, unknown	M, F, unknown	M, F	M, F	M, F, unknown	M, F	M, F, unknown
Age (years)	44 to 98, unknown	51 to 98, unknown	68 to 77	55 to 89	51 to 93, unknown	58 to 87	69 to 91
Bone quality	Normal, osteopenic, osteoporotic, unknown	Normal, osteopenic, osteoporotic, unknown	Osteoporotic, unknown	Normal, osteopenic, osteoporotic, unknown	Normal, osteoporotic	Osteoporotic	Osteoporotic, unknown
Fracture level	T3, T6 to L5	T2 to L5, unknown	T2 to L5	T11 to L5	T6 to L5, unknown	T10, L1, L2	L1, L4, unknown
Fracture type	Wedge, unfractured	Wedge, burst, unfractured	Wedge, burst	Wedge	Wedge	Wedge	Wedge, unfractured
Test set-up	Parallel plates, 1 pivoting plate, 6DOF tester	Parallel plates, 1 pivoting plate, 2 pivoting plates, 6DOF tester	Parallel plates, 1 pivoting plate	1 pivoting plate, 2 pivoting plates, 6DOF tester	1 pivoting plate	2 pivoting plates	Parallel plates, 1 pivoting plate, 6DOF tester
Load type	Quasi-static, cyclic	Quasi-static, cyclic	Quasi-static	Quasi-static, cyclic	Quasi-static, cyclic	Quasi-static, cyclic	Quasi-static, cyclic
Stiffness (N/mm)	200 - 4605	134 - 3863	894 - 7000	323 - 1490	77 - 138	404 - 982	2500
Failure (N)	3584 - 7832	1167 - 5703	2473 - 11,400	4672 - 4702	767 - 5088	—	3900 - 4250
VH (% of intact)	47 - 89	57 - 108	90	62 - 113	83 - 102	94	—
Other secondary outcomes	CL, CS, CV, IDP, NF, NS, ROM	BS, CL, CV, IDP, IR, KA, NF, NS, ROM	CV, KA, NF, NS	CV, KA, ROM	CV, IR, KA	CV	CV, ROM

BS: bone strain; CL: cement leakage; CS: contact stress at loading plate/vertebra interface; CV: cement volume required; F: female; IDP: internal disc pressure; IR: implant rotation; KA: kyphosis angle; M: male; NF: normalized failure; NS: normalized stiffness; ROM: range of motion; 6DOF: 6-degree-of-freedom.

## Data Availability

Data is not applicable.
